# Effect of Hypertension on Bone Mineral Density of Patients with Rheumatoid Arthritis

**DOI:** 10.31138/mjr.120923.eoh

**Published:** 2023-09-12

**Authors:** Praveen Pratap Jadhav, Vivek Gajanan Patwardhan

**Affiliations:** 1 Omkar Rheumatology Clinic, Gaikwad Mala, Nashik, India,; 2 Hirabai Cowasji Jehangir, Medical Research Institute, Jehangir Hospital, Pune, India

**Keywords:** osteoporosis, bone mineral density, hypertension, T score

## Abstract

**Objective:**

Patients with rheumatoid arthritis (RA) are associated with low bone mineral density (BMD). Chronic comorbidities such as type II diabetes mellitus have shown to affect BMD parameters in patients with RA. Hypertension (HT) is a chronic disease and its coexistence with RA can alter bone health. The aim of this study was to investigate if HT affected BMD parameters in RA patients diagnosed for the first time.

**Methods:**

Patients with the diagnosis of RA who underwent BMD studies formed the study population. Patients with HT were sorted from this population and formed a separate group. Healthy controls were drawn from subjects who came for a check-up. BMD was done with the GE Lunar DPX machine. Mean T Scores at spine, femur neck and total femur were recorded. Data from the three groups were analysed and compared. Linear regression analyses were performed.

**Results:**

Analysis suggested that the age had inverse and BMI had direct correlation with BMD T scores in all groups. The additional diagnosis of HT in RA patients was associated with higher BMD as compared to patients with RA, but lower than controls. R^2^ values were 0.341, 0.402 and 0.436 for mean T scores at spine, femur neck and femur total respectively. Figures from multiple regression analysis suggest that BMI alone did not explain the higher T score values in HT patients.

**Conclusion:**

Additional morbidity of HT in RA patients negates the porotic effect of RA as judged by bone densitometry. Hence, BMD reports should be read with caution in these patients.

## INTRODUCTION

Rheumatoid Arthritis (RA) is a chronic inflammatory disease which affects various organs. Bone is an important organ affected by RA. It is manifested as low bone mineral density (BMD),^1,2^ generalised osteoporosis,^1,2^ and increased risk of fragility fractures.^3,4^ RA is a key factor determining the risk of fractures in the assessment of Fracture Risk Assessment Score (FRAX) score.^5^ Hypertension (HT) is also a chronic disease which can affect multiple systems. The effect of hypertension on bone health is controversial. While many studies have shown that HT has a negative effect,^6,7^ some have shown none,^8,9^ while a few others have shown a positive effect in men on BMD^10^. In spite of variable effect of HT on BMD, it has been strongly suggested that HT is an independent risk factor for osteoporosis and osteoporotic fractures in men and postmenopausal women.^7,11^

The effect of coexisting HT with RA on BMD has not been well studied. Associated comorbidities like type II diabetes mellitus (T2DM) have shown to alter BMD parameters in RA patients.^12^ It is possible that HT, by the virtue of its effects mentioned above, can alter BMD values in these patients. The aim of this study was to investigate if HT affected BMD parameters in newly diagnosed RA patients. We also studied if variables such as age or body mass index confounded the results.

## MATERIALS AND METHODS

This was a single-centre cross-sectional study. The centre has a database of patients following over the last 5 years. Patients with the diagnosis of RA who underwent BMD studies were included in the study. The American College of Rheumatology Criteria (ACR) 2019 were used as a guide to diagnose RA. Since RA is a risk factor for osteoporosis, all patients presenting to the centre were advised DXA scan. Though patients presented after variable duration of symptoms, they were diagnosed as RA for the first time. Hence all these patients were treatment naïve for DMARDS. The mean Disease Activity Score of these patients at presentation was not significantly different in patients with RA and RA with HT and is mentioned in **Table 1**. **Table 1** also shows the demographics and associated comorbidities of the study population. Patients with HT were separated from this population and formed a separate group. These patients were diagnosed as hypertensives prior to their diagnosis of RA by their primary physicians based on their blood pressure readings of more than 140/90 mmHg. All these patients were on medications.

**Table 1.** Comorbidities associated with the study population.

		**RA**	**RA with HT**	**Total**	
	**N**	**396**	**156**	**552**	**Stat: chi square**
**Smoking**	NO	388 (97.98%)	149 (95.51%)	537 (97.28%)	Χ^2^ (1, N=552)= 2.576, p= 0.108
	YES	8 (2.02%)	7 (4.49%)	15 (2.72%)	
**Tobacco**	NO	373 (94.19%)	145 (92.95%)	518 (93.84%)	Χ^2^ (1, N=552)= 0.299, p= 0.584
	YES	23 (5.81%)	11 (7.05%)	34 (6.16%)	
**Alcohol**	NO	390 (98.48%)	152 (97.44%)	542 (98.19%)	Χ^2^ (1, N=552)= 0.692, p= 0.405
	YES	6 (1.52%)	4 (2.56%)	10 (1.81%)	
**Diabetes**	NO	357 (90.15%)	138 (88.46%)	495 (89.67%)	Χ^2^ (1, N=508)= 4.188, p= 0.041
	YES	6 (1.52%)	7 (4.49%)	13 (2.36%)	

Age-matched controls were drawn from healthy volunteers who came for a routine annual health checkup and who agreed to use their data for the study. These were healthy subjects from same geographical area, with no comorbidities and no addictions. The controls were matched with the study population for age, but as they were healthy subjects, comorbidities were not present and were not intended to match for comorbidities. Notably, the BMI of controls was significantly better than the study population by the virtue of them being healthy. The details of these healthy volunteers have been published in a previous study. ^13^

BMD was done with the GE Lunar DPX machine. Mean T Scores at spine, femur neck, and total femur were recorded. T Scores rather than actual bone densities were used for analysis to give unequivocal picture to the readers, and also because, more recently, many publications are using it as preferable way to convey the BMD.^14,15^ Two technicians performed equal number of studies randomly throughout the period of 5 years, minimising operator dependant variability.

Data were analysed using the SPSS software for Windows (version 26.0, IBM Corporation, USA). Normality of the variables was tested using skewness, kurtosis, one sample Kolmogorov-Smirnov test and Shapiro-Wilk test before performing statistical analysis. Levene’s test was used to test homogeneity of variance**.** Continuous variables were presented as mean with standard deviation. In the entire study, the p-values less than 0.05 were considered statistically significant. All the hypotheses were formulated using two tailed alternatives against each null hypothesis (hypothesis of no difference). Oneway ANOVA was used to examine differences in mean of variables between groups. Kruskal-Wallis test was used for non-normal data. Welsh correction was used for non-homogeneous data. Multiple stepwise linear regression was performed to assess the influence of independent variables on dependent variables. Regression assumptions co-linearity, normality and homoscedasticity were assessed using VIF, P-P plots and scatter plot of residuals, respectively.

## RESULTS

A total of 552 patients (M-85, F-467) diagnosed as RA who had their BMD examined were enrolled in the study. Of these, 156 (M-28, F-128) patients had HT. Five hundred three (M-250, F-253) healthy controls were enrolled from routine health check-ups. As a group, the controls were age matched with patients in the study. The DAS scores in patients with RA and RA with coexisting HT were not significantly different both in females and males (*p*=>0.05) as measured by the unpaired t test (**Table 2**). Chi square analysis showed that addictions were also not significantly different in these two groups (**Table 1**). Diabetes was significantly higher in RA with HT patients (4.49%) versus RA patients (1.52%). However, since the percentage of diabetic population was quite low, it is unlikely to affect the overall BMD values of the study population.

**Table 2.** T Scores at different sites in controls, patients with RA and patients with RA+HT.

	**Female**			
	**Controls**	**RA**	**RA with HT**	**Total**
**N**	n = 250	n = 339	n = 128	n = 717
**Mean Age**	54.05 ± 8.63	52.67 ± 11.65	57.59 ± 13.11	54.03 ± 11.12
**Mean BMI ^a , b , c^**	27.84 ± 4.76	22.36 ± 4.52	26.66 ± 5.3	25.05 ± 5.39
**Mean T Score Femur neck ^a , b , c^**	-1.13 ± 0.93	-2.5 ± 0.94	-1.85 ± 1.04	-1.91 ± 1.13
**Mean T Score femur total ^a , b , c^**	-0.63 ± 1.05	-2.28 ± 0.89	-1.54 ± 1.51	-1.57 ± 1.31
**Mean T Score Spine ^a , b , c^**	-1.35 ± 1.42	-2.98 ± 1.16	-2.27 ± 1.34	-2.28 ± 1.48
**DAS scores**	-	5.38 ± 1.64	5.12 ± 1.55	
	**Male**			
	**Controls**	**RA**	**RA with HT**	**Total**
**N**	n = 253	n = 57	n = 28	n = 338
**Mean Age**	54.98 ± 10.48	56.35 ± 12.75	59.61 ± 11.81	55.59 ± 11.05
**Mean BMI ^a , c^**	26.31 ± 3.67	21.42 ± 3.49	26.41 ± 4.17	25.49 ± 4.11
**Mean T Score Femur neck ^a , c^**	-1.19 ± 0.96	-2.07 ± 0.82	-1.42 ± 1.16	-1.36 ± 1
**Mean T Score femur total ^a , c^**	-0.88 ± 0.87	-1.96 ± 0.88	-1.1 ± 0.94	-1.08 ± 0.96
**Mean T Score Spine ^a , c^**	-1.09 ± 1.27	-2.36 ± 1.9	-1.59 ± 1.16	-1.34 ± 1.46
**DAS scores**	-	4.81 ± 1.64	4.98 ± 1.81	-

a significantly different between control and RA;

b significantly different between control and RA with HT;

c significantly different between RA and RA with HT.

A separate analysis was done for men and women with age matched controls.

Analysis of variance (ANOVA) tests were conducted with age, BMI, mean T scores at femur neck, mean T scores for Femur total and mean T scores for spine as dependent parameters and diagnosis (controls, RA and RA with hypertension) as fixed factor to understand differences between the groups.

**Figure 1.**
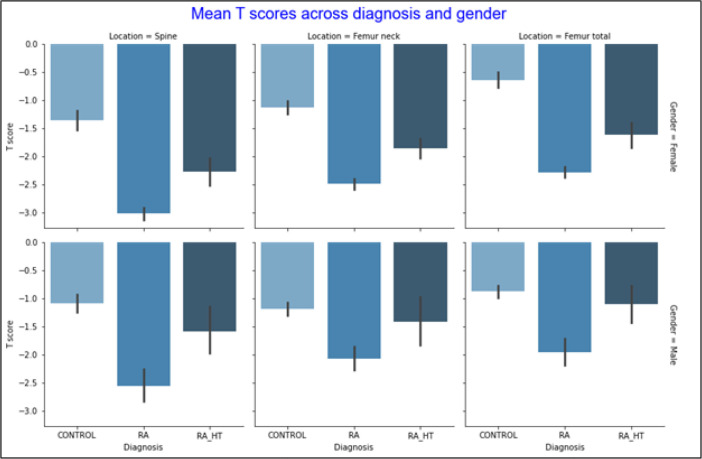
KDE plots of T Scores versus density at different sites in controls, patients with RA and patients with RA+HT.

**Figure 2.**
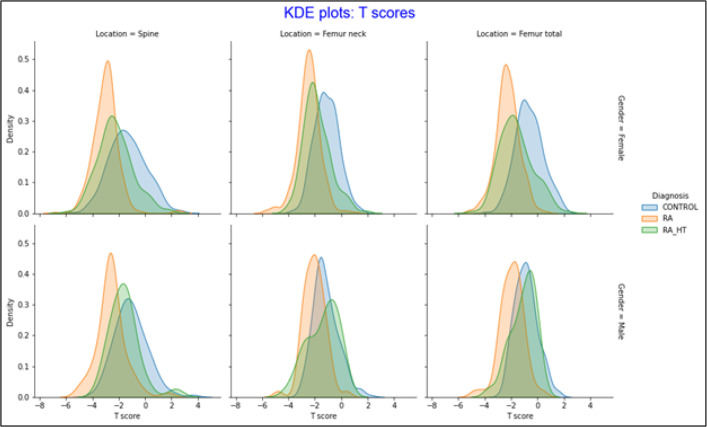
Scatter plot of BMI versus T Scores at different sites in controls, patients with RA and patients with RA+HT

ANOVA results indicated that in females, overall significant difference was seen in BMI [F (2, 711) = 104.492, p<0.05], Mean T scores Femur neck [F (2, 713) = 149.878, p<0.05], Mean T scores Femur total [F (2, 700) = 164.243, p<0.05] and Mean T scores Spine [F (2, 703) = 113.566, p<0.05] between controls, patients with RA and patients of RA with coexisting HT (**Table 2**). In males, overall significant difference was seen in Mean BMI [F(2, 335)=41.935, p<0.05], Mean T scores Femur neck [F(2, 335)=20.128, p<0.05], Mean T scores Femur total [F(2, 333)=34.538, p<0.05], Mean T scores Spine [F(2, 331)=19.579, p<0.05] between controls, patients with RA and patients of RA with coexisting HT (**Table 2**). The mean values of T scores at different sites are shown in **Figures 1** and **2**.

Multiple linear regression analysis was done with T scores as dependent and age, BMI, RA, RA with HT as independent parameters for females and males. Linear regression analysis suggested that the age had inverse and BMI had direct correlation with BMD T scores in all groups. The analysis also suggested that the diagnosis of HT was also associated with higher BMD. Figures from multiple regression analysis suggest that BMI alone did not explain the higher T score values in HT patients (**Table 3**).

**Table 3.** Summary of multiple linear regression.

**Dependent parameter**	**T Scores Spine**		**T Scores Femur Neck**		**T Scores Femur Total**	
	**Female**						
**Model R^2^**	R^2^=0.341		R^2^=0.402		R^2^=0.436	
	Beta	p	Beta	p	Beta	p
**Age**	-0.267	< 0.05	-0.253	< 0.05	-0.228	< 0.05
**BMI**	0.192	< 0.05	0.252	< 0.05	0.297	< 0.05
**RA**	-0.47	< 0.05	-0.492	< 0.05	-0.492	< 0.05
**RA with HT**	-0.188	< 0.05	-0.192	< 0.05	-0.214	< 0.05
	**Male**						
**Model R^2^**	R^2^=0.156		R^2^=0.22		R^2^=0.252	
	Beta	p	Beta	p	Beta	p
**Age**	-	0.102	-0.164	< 0.05	-	0.785
**BMI**	0.251	< 0.05	0.326	< 0.05	0.334	< 0.05
**RA**	-0.213	< 0.05	-0.171	< 0.05	-0.261	< 0.05
**RA with HT**	-0.089	< 0.05	-	0.341	-	0.181

DAS was not significantly different between patients with RA and RA+HT.

## DISCUSSION

That RA predisposes to bone loss is well known and is confirmed by this study. However, more importantly, this study suggests that the additional comorbidity of HT partially negates the effect of RA on BMD. The BMD values of hypertensive patients with RA are significantly better than patients with RA, but lower than the controls. Subset analysis based on gender showed only minor differences as compared to the whole group. The behaviour of T scores in the female group was similar to the behaviour of the total population. This was expected since women formed 82.5% of the population. In males with HT and RA, the T scores at spine and total femur were significantly better than in males with RA alone but were not significantly different from the controls. This minor deviation on the male subset as compared to the entire group could be due to the smaller number of males in the group.

RA is well known to cause bone loss reflecting it in lower BMD values.^1,4,16,17^ This study confirms this previously known fact. The incidence of HT is rising.^18^ As in the general population, it’s incidence in rheumatologic diseases is also expected to rise proportionately. Hence, physicians should be aware of how these coexisting morbidities like HT can affect bone density.

The reports of effects HT alone (without RA) on BMD have been inconsistent. While a few studies have shown a positive effect of HT on BMD,^19^ few have shown no effect^20,21^ and some negative effect on BMD.^22,23^ In a meta-analysis of nine studies, five studies suggested that hypertensives had lower BMD than non-hypertensive controls, while four studies suggested that hypertension and BMD were unrelated.^7^

We did not find any study examining the effects of HT on BMD in RA patients. To the best of our knowledge, this is the first study to suggest that HT negates the effect of RA on BMD.

Patients with hypertension and RA had a significantly higher BMI as compared to patients with RA alone. BMI has already shown to have positive correlation with BMD parameters. ^24,25^This could be one mechanism by which the hypertensive patients in the group had higher BMD parameters. However, in depth analysis showed that this was not the only factor responsible for better BMD parameters in hypertensive patients.

The effect of hypertension on bones seems to be regulated through the renin angiotensin aldosterone (RAAS) axis. While animal studies suggest that RAAS stimulation has positive effects on bone metabolism,^26^ human studies have shown varied results. The RAS was found to be active locally in the inflamed synovial tissue and ACEI was presumed to be helpful in reducing bone loss.^27^ Albeit this, and more importantly, clinical studies have shown opposite results. Use of ACE inhibitors was suggested to increase bone loss in elderly American men.^28^ Continuous use of ACE inhibitors for more than 4 years in elderly Chinese women was associated with increased bone loss in total hip and femoral neck.^29^ In the Japanese Adult Health Study, patients taking long term ACE inhibitors had an annual decline of 0.61% in their BMD values even after adjustment of confounding factors in their biennial follow ups.^30^ In a study of post-menopausal women by Carbone et al, use of RAAS blockers, both ACE inhibitors and ARBs, have been associated with increased risk of fragility fractures at least in the first 3 years.^31^Action of ACE inhibition in turn could be mediated through its effect on inhibition of dehydroepiandrosterone (DHEA) production. DHEA is a sex hormone which has an important role in bone anabolism. ACE inhibitor use was associated with significantly lower serum DHEA levels in older men.^32,33^ The role of RAAS activation has already been suggested and elaborated in the pathogenesis of RA by Moeriera et al.^34^ In summary, evidence of high RAAS in RA patients and deleterious effects of RAAS inhibitors on bone density suggests that hypertension may negate bone loss associated with RA through this mechanism too.

Does hypertension predispose to fragility fractures? The association between hypertension and BMD is still unclear. A case control study including 124,655 fracture cases and 373,962 age- and gender-matched controls suggested that hypertension was associated with a 1.2-fold increase in risk of fractures.^35^ Another Swedish population-based study suggested that hypertension increased the multivariable-adjusted hip fracture risk.^36^ Recent data from the Dubbo Osteoporosis Epidemiology Study indicated that a positive relationship between hypertension and fracture risk, in women but not in men, which however is independent of BMD.^21^ In the current fracture risk assessment models (FRAX® or Garvan Bone Fracture Risk Calculator), hypertension is still not a recognized risk factor for osteoporotic fracture largely because of a lack of prospective studies. Higher BMI is associated with stronger bones. Since this is one likely mechanism of higher BMD values in hypertensive patients, it is possible that the bone quality in this subset is indeed better and therefore may result in fewer fractures. This is unlike in T2DM, where in spite of higher BMD values, the bone quality is postulated to be poor.^12^ Hence, prospective studies are needed to provide a definitive answer about the significance of better BMD values in hypertensive patients with RA to determine its impact on fracture risk.

The higher measures could mean a true improvement in the quality of the bone. Alternatively, it could also represent falsely elevated values of a weaker bone. Like in type II DM, falsely elevated BMD values could be due to the difference in cortical and medullary bone densities.^37,38^ Hence, till the time future studies throw a light about the mechanisms and till we have results from prospective studies of fracture risk in hypertensive RA patients, the BMD results read by DEXA scan in hypertensive patients would be subject to suspicion. Also, in research studies, addition of HT as comorbidity in RA patients could alter the BMD results.

There are a few limitations to the study. Firstly, though the relation of hypertension and BMD could be evaluated, its relationship with the severity and duration of hyper-tension could not be done. Secondly, it is possible that antihypertensive medications could have altered some values of BMD. The role of anti-hypertensive medications could not be ascertained from our data.

## CONCLUSION

This study suggests that as compared to RA patients, coexisting HT and RA present with significantly higher BMD measures. BMD as measured by DEXA scan in RA patients could be compromised and these measures, in this population should be read with caution. Whether the improved BMD measures in these patients translate into lower risk of fragility fractures needs to be investigated.

## NOTE

The corresponding author is the Director of the Centre where the patients were examined, data was archived and later analysed. He has the authority and has consented for the analyses of archived data. No help from external editing agencies was obtained for the preparation of the manuscript. Both the authors take full responsibility of the authenticity, accuracy, and integrity of the data.

## References

[1] Ruaro B Lems W Pino J Del González-Álvaro I Castañeda S Llorente I Osteoporosis in Rheumatoid Arthritis: Dangerous Liaisons. Front Med 2020;7:601618. 10.3389/fmed.2020.601618. PMC7719815 33330566

[2] Pouresmaeili F Kamali Dehghan B Kamarehei M Yong Meng G. A comprehensive overview on osteoporosis and its risk factors. Ther Clin Risk Manag 2018 ; Volume 14 : 2029 – 49 . 10.2147/TCRM.S138000 . PMC6225907 30464484

[3] Cooper C Coupland C MM. Rheumatoid arthritis, corticosteroid therapy and hip fracture. Ann Rheum Dis 1995;54:49–52. 10.1136/ard.54.1.49. 7880122 PMC1005512

[4] Huusko TM Korpela M Karppi P Avikainen V Kautiainen H Sulkava R. Threefold increased risk of hip fractures with rheumatoid arthritis in central Finland. Ann Rheum Dis 2001;60:521–2. 10.1136/ard.60.5.521. 11302878 PMC1753630

[5] Kanis JA Johnell O Oden A Johansson H McCloskey E. FRAX^TM^ and the assessment of fracture probability in men and women from the UK. Osteoporos Int 2008;19:385–97. 10.1007/s00198-007-0543-5. 18292978 PMC2267485

[6] Cappuccio FP Meilahn E Zmuda JM Cauley JA. High blood pressure and bone-mineral loss in elderly white women: a prospective study. Lancet 1999;354:971–5. 10.1016/S0140-6736(99)01437-3. 10501357

[7] Yang Wu. Osteoporosis and Osteoporotic Fracture: Contribution of Hypertension and Anti-hypertension Medications. Austin J Clin Med 2014;11. Yang,:1009.

[8] Javed F Khan SA Ayers EW Aziz EF Akram MS Nadkarni GN Association of hypertension and bone mineral density in an elderly African American female population. J Natl Med Assoc 2012;104:172–8. 10.1016/S0027-9684(15)30140-1. 22774384

[9] Zhang M Yagang L Ying L Xuena P Binbin L Zhong G Study on the influencing factors for bone mineral density among 24831 people in Changchun. Chinese J Osteoporos 2012;2:125–7.

[10] Yang TT Xiao-Hong LV RF. Intervention study and influential factors of elder osteoporotic fracture. Mod Prev Med 2012;39:

[11] Li C Zeng Y Tao L Liu S Ni Z Huang Q Meta-analysis of hypertension and osteoporotic fracture risk in women and men. Osteoporos Int 2017;28:2309–18. 10.1007/S00198-017-4050-Z/FIGURES/5. 28447105

[12] Jadhav P Patwardhan V. Effect of type 2 diabetes mellitus on bone mineral density in patients with rheumatoid arthritis. Indian J Rheumatol 2021;16:276. 10.4103/INJR.INJR_293_20.

[13] Kadam NS Chiplonkar SA KA VV. K. Prevalence of osteoporosis in apparently healthy adults above 40 years of age in Pune City, India. Indian J Endocr Metab 2018;22:67–73. 10.4103/ijem.IJEM_438_17 PMC5838914 29535940

[14] C Libanati CJFL J Malouf-Sierra JR. Relationship Between Bone Mineral Density T-Score and Nonvertebral Fracture Risk Over 10 Years of Denosumab Treatment. JBMR 2019;34:1033–40. 10.1002/jbmr.3722 PMC6852155 30919997

[15] Cummings SR Martin JS Mcclung MR Siris ES Eastell R Reid IR Denosumab for Prevention of Fractures in Postmenopausal Women with Osteoporosis 2009. 10.1056/NEJMoa0809493. 19671655

[16] Peng J Gong Y Zhang Y Xiao Z Zeng Q Chen S. Bone mineral density in patients with rheumatoid arthritis and 4-year follow-up results. J Clin Rheumatol 2016;22:71–4. 10.1097/RHU.0000000000000359. 26906298

[17] Lodder MC De Jong Z Kostense PJ Molenaar ETH Staal K Voskuyl AE Bone mineral density in patients with rheumatoid arthritis: Relation between disease severity and low bone mineral density. Ann Rheum Dis 2004;63:1576–80. 10.1136/ard.2003.016253. 15547081 PMC1754831

[18] Mills KT Stefanescu A He J. The global epidemiology of hypertension n.d. 10.1038/s41581-019-0244-2. PMC7998524 32024986

[19] Hanley DA Brown JP Tenenhouse A Olszynski WP Ioannidis G Berger C Associations Among Disease Conditions, Bone Mineral Density, and Prevalent Vertebral Deformities in Men and Women 50 Years of Age and Older: Cross-Sectional Results From the Canadian Multicentre Osteoporosis Study*. 2003. 10.1359/jbmr.2003.18.4.784 12674340

[20] Hijazi N Alourfi Z. Association between Hypertension, Antihypertensive Drugs, and Osteoporosis in Postmenopausal Syrian Women: A Cross-Sectional Study. Adv Med 2020;2020:1–6. 10.1155/2020/7014212. PMC7049845 32149161

[21] Yang S Nguyen ND Center JR Eisman JA Nguyen T V. Association between hypertension and fragility fracture: a longitudinal study. Osteoporos Int 2014;25:97–103. 10.1007/s00198-013-2457-8. 23892585

[22] Yazici S Yazici M Korkmaz U Erkan ME Erdem Baki A Erden I Relationship between blood pressure levels and bone mineral density in postmenopausal Turkish women 2011. https://doi.org/10.5114/aoms.2011.22077. Arch Med Sci 2011 Apr;7(2):264–70. 10.5114/aoms.2011.22077. 22291766 PMC3258724

[23] Ye Z Lu H Liu P. Association between essential hypertension and bone mineral density: a systematic review and meta-analysis. vol. 8. 2017. 10.18632/oncotarget.20325 PMC5620307 28978167

[24] Salamat MR Salamat AH Abedi I Janghorbani M. Clinical Study Relationship between Weight, Body Mass Index, and Bone Mineral Density in Men Referred for Dual-Energy X-Ray Absorptiometry Scan in Isfahan, Iran. J Osteoporos 2013;2013:. 10.1155/2013/205963. PMC3814102 24222888

[25] Hoxha R Islami H Qorraj-Bytyqi H Thaçi S Bahtiri E. Relationship of Weight and Body Mass Index with Bone Mineral Density in Adult Men from Kosovo. Mater Sociomed 2014. 10.5455/msm.2014.26.306-308. PMC4272829 25568627

[26] Akagi T Mukai T Mito T Kawahara K Tsuji S Fujita S Effect of Angiotensin II on Bone Erosion and Systemic Bone Loss in Mice with Tumor Necrosis Factor-Mediated Arthritis. Int J Mol Sci 2020;21:1–19. 10.3390/IJMS21114145. PMC7312645 32532031

[27] Zhao J Yang H Chen B Zhang R. The skeletal renin-angiotensin system: A potential therapeutic target for the treatment of osteoarticular diseases. Int Immunopharmacol 2019;72:258–63. 10.1016/J.INTIMP.2019.04.023. 31003003

[28] Kwok T Leung J Zhang YF Ensrud KE Barrett-Connor E Leung PC. Does the use of ACE inhibitors or angiotensin receptor blockers affect bone loss in older men? 2011. 10.1007/s00198-011-1831-7. PMC3772278 22080379

[29] Zhang YF Qin L Leung PC Kwok TCY. The effect of angiotensin-converting enzyme inhibitor use on bone loss in elderly Chinese. J Bone Miner Metab 2012;30:666–73. 10.1007/S00774-012-0363-3. 22743851

[30] Masunari N Fujiwara S Nakata Y Furukawa K Kasagi F. Effect of angiotensin converting enzyme inhibitor and benzodiazepine intake on bone loss in older Japanese. Hiroshima J Med Sci 2008;57:17–25. 18578363

[31] Carbone LD Vasan S Prentice RL Harshfield G Haring B Cauley JA The renin-angiotensin aldosterone system and osteoporosis: findings from the Women’s Health Initiative. Osteoporos Int 2019 3010 2019;30:2039–56. 10.1007/S00198-019-05041-3. 31209511

[32] Kwok T Ohlsson C Vandenput L Tang N Zhang YF Tomlinson B ACE inhibitor use was associated with lower serum dehydroepiandrosterone concentrations in older men. Clin Chim Acta 2010;411:1122–5. 10.1016/J.CCA.2010.04.011. 20403346 PMC2883618

[33] Kirby DJ Buchalter DB Anil U Leucht P. DHEA in bone: the role in osteoporosis and fracture healing. Arch Osteoporos 2020;15:84. 10.1007/s11657-020-00755-y. 32504237

[34] Moreira FRC de Oliveira TA Ramos NE Abreu MAD Simões e Silva AC. The role of renin angiotensin system in the pathophysiology of rheumatoid arthritis. Mol Biol Rep 2021;48:6619–29. 10.1007/s11033-021-06672-8. 34417705

[35] Peter Vestergaard LR& LM. Hypertension Is a Risk Factor for Fractures. Calcif Tissue Int 2009;84:103–11. 19067019 10.1007/s00223-008-9198-2

[36] Sennerby U. Farahmand B. SL& KM A. Ahlbom. Cardiovascular diseases and future risk of hip fracture in women. Osteoporos Int 2007;18:1355–62. 17492247 10.1007/s00198-007-0386-0

[37] Ho-Pham LT Chau PMN Do AT Nguyen HC Nguyen T V. Type 2 diabetes is associated with higher trabecular bone density but lower cortical bone density: the Vietnam Osteoporosis Study. Osteoporos Int 2018;29:2059–67. 10.1007/s00198-018-4579-5. 29967929

[38] Melton LJ Riggs BL Leibson CL Achenbach SJ Camp JJ Bouxsein ML A bone structural basis for fracture risk in diabetes. J Clin Endocrinol Metab 2008;93:4804–9. 10.1210/jc.2008-0639. 18796521 PMC2626440

